# Novel Antimicrobial Peptides That Inhibit Gram Positive Bacterial Exotoxin Synthesis

**DOI:** 10.1371/journal.pone.0095661

**Published:** 2014-04-18

**Authors:** Joseph A. Merriman, Kimberly A. Nemeth, Patrick M. Schlievert

**Affiliations:** 1 Department of Microbiology, Carver College of Medicine, University of Iowa, Iowa City, Iowa, United States of America; 2 The Procter & Gamble Company, Cincinnati, Ohio, United States of America; National Institutes of Health, United States of America

## Abstract

Gram-positive bacteria, such as *Staphylococcus aureus*, cause serious human illnesses through combinations of surface virulence factors and secretion of exotoxins. Our prior studies using the protein synthesis inhibitor clindamycin and signal transduction inhibitors glycerol monolaurate and α-globin and β-globin chains of hemoglobin indicate that their abilities to inhibit exotoxin production by *S. aureus* are separable from abilities to inhibit growth of the organism. Additionally, our previous studies suggest that inhibition of exotoxin production, in absence of ability to kill *S. aureus* and normal flora lactobacilli, will prevent colonization by pathogenic *S. aureus*, while not interfering with lactobacilli colonization. These disparate activities may be important in development of novel anti-infective agents that do not alter normal flora. We initiated studies to explore the exotoxin-synthesis-inhibition activity of hemoglobin peptides further to develop potential agents to prevent *S. aureus* infections. We tested synthesized α-globin chain peptides, synthetic variants of α-globin chain peptides, and two human defensins for ability to inhibit exotoxin production without significantly inhibiting *S. aureus* growth. All of these peptides were weakly or not inhibitory to bacterial growth. However, the peptides were inhibitory to exotoxin production with increasing activity dependent on increasing numbers of positively-charged amino acids. Additionally, the peptides could be immobilized on agarose beads or have amino acid sequences scrambled and still retain exotoxin-synthesis-inhibition. The peptides are not toxic to human vaginal epithelial cells and do not inhibit growth of normal flora *L. crispatus*. These peptides may interfere with plasma membrane signal transduction in *S. aureus* due to their positive charges.

## Introduction

*Staphylococcus aureus* is a gram-positive bacterium that is a significant cause of disease throughout the world. The organism is ubiquitous, with estimates of almost 40% of humans being colonized on mucosal surfaces [1], [2]. The illnesses caused by the organism range from relatively benign infections such as furuncles and soft tissue abscesses, to life-threatening illnesses such as toxic shock syndrome (TSS), pneumonia, sepsis, and infective endocarditis [1]. *S. aureus* causes diseases through production of a large number of cell surface and secreted virulence factors [1], [2]. One of the major secreted exotoxins is the superantigen TSS toxin-1 (TSST-1) [3]–[5]. TSST-1 is the principal cause of menstrual TSS, a condition typically associated with healthy women who are using tampons, colonized vaginally with *S. aureus*, and unable to produce antibody responses to the superantigen [6]–[10]. Additionally, TSST-1 is the cause of up to 50% of non-menstrual TSS, with most cases being associated with upper respiratory tract infections; most of the remaining non-menstrual TSS is associated with the superantigens staphylococcal enterotoxins B and C [6], [7], [10]. Superantigens cause serious human illnesses by causing massive cytokine production, resulting in an acute-onset illness characterized by fever and vomiting and diarrhea (flu-like symptoms), hypotension, a sunburn-like rash, peeling of the skin upon recovery, and a variable multi-organ component [2], [10]–[13].

We have previously shown that when *S. aureus* is grown in the presence of human blood, such as would be present in tampons during menstruation, TSST-1 and cytolysin production is significantly decreased or completely inhibited through hemoglobin chain actions on one or more bacterial two component systems, especially SrrA/B and Agr A/C [14], despite otherwise favorable conditions for exotoxin production (37°C, neutral pH, medium with protein, ≥2% O_2_, and 7% CO_2_) [15], [16]. These studies suggest the mechanism of hemoglobin chain action was to inhibit transcription of exotoxin genes. In 1984, we also showed that the antibiotic clindamycin inhibits superantigen production at concentrations that fail to inhibit bacterial growth [17]. We have recently shown that two other compounds, glycerol monolaurate and chitosan, also inhibit TSST-1 production at concentrations that do not inhibit staphylococcal growth [18], [19]. These molecules also interfere with plasma membrane signal transduction [18]–[21]. Collectively, our studies suggest that exotoxin production by *S. aureus* is separable from growth of the organism, and agents can be found that uniquely target the cell surface to prevent exotoxin production while not killing the organism. It would be expected that these agents also will not affect the growth of normal mucosal lactobacilli [22], as has been shown in studies of GML [23]–[25].

Exotoxin-synthesis-inhibition by peptides may prevent microbial infections on human mucosal surfaces by organisms like *S. aureus* that depend on exotoxin production. Our recent studies suggest that *S. aureus* and streptococci produce exotoxins that initiate low-level host inflammatory responses from epithelial cells, and this facilitates their colonization [26]. Additionally, our recent studies to vaccinate rabbits against *S. aureus* exotoxins prevented pulmonary colonization, further suggesting that exotoxin production by this organism was critical for colonization [27], [28].

The present study was undertaken to evaluate both naturally occurring hemoglobin-derived peptides and a variety of synthetic derivatives for their abilities to inhibit TSST-1 and α-toxin production while concurrently minimally affecting *S. aureus* growth. Our studies with hemoglobin peptides show that these molecules inhibit TSST-1 and α-toxin production without major inhibitory effects on *S. aureus* and normal flora *Lactobacillus crispatus* growth. This observation allowed us to design and test additional peptides that exhibited even greater activity than the original hemoglobin peptides without affecting microbial growth.

## Materials and Methods

### Bacterial growth studies

*S. aureus* MN8 and MNPE, a menstrual TSS isolate and fatal post-influenza pulmonary TSS isolate, respectively, were used for all experimentation [5]; strain MN8 is representative of approximately 75% of menstrual TSS isolates. The organisms are classified by the Centers for Disease Control and Prevention as USA200 by pulsed-field gel electrophoresis. *S. aureus* MN8 is known to produce approximately 20 µg/ml of TSST-1 in broth cultures [17], whereas MNPE produces approximately 5 µg/ml of TSST-1 and approximately 50 µg/ml of α-toxin [29]. The organisms are maintained in the Schlievert laboratory in the lyophilized state. For experimentation, MN8 and MNPE were cultured overnight in Todd Hewitt (Difco laboratories, Detroit, MI). The next day, the organisms were diluted in fresh Todd Hewitt broth for inoculation (final inocula were approximately 10^4^/ml). For all peptide studies, *S. aureus* MN8 and MNPE were cultured for 9 h with shaking (200 revolutions/min) in the presence of potential antimicrobial peptides (5×10^−6^ µg/ml to 5.0 µg/ml) in volumes of 2 ml culture per tube. After incubation, a sample of each culture was used for plate-count determination of CFUs/ml, and a sample was used for TSST-1 quantification (MN8) or TSST-1 and α-toxin quantification (MNPE) [14].

For TSST-1 measurement, 1 ml of each sample (cells plus culture fluid) was treated overnight with 4 volumes of absolute ethanol; we have previously shown that this treatment precipitates all measurable TSST-1 [15]. Subsequently, the precipitate from each culture was collected by centrifugation (4000×g, 10 min), ethanol decanted, and sample dried for 30 min under a laminar flow hood. Each sample was resuspended in distilled water (100 µl) and clarified by centrifugation (14,000×g, 5 min). We have also previously shown that intact TSST-1 cannot be detected intracellularly in *S. aureus*, even under optimal production conditions, consistent with TSST-1 having a signal peptide required for TSST-1 transport and folding of the protein into native structure outside the bacterial plasma membrane. This was done through internal radiolabeling (^35^S methionine) to 2×10^10^ disintegrations/min per microgram of secreted TSST-1 [30]. Subsequently, the bacterial cells were treated with lysostaphin in isotonic solution to remove cell walls, washed, and lysed with osmotic shock [31]. No TSST-1 could be detected after subsequent ethanol precipitation and Western immunoblot (described below). This procedure, combined with demonstration of complete precipitation of TSST-1 with 4 volumes of ethanol, validated our method to concentrate TSST-1.

TSST-1 production by strain MN8 was determined through a quantitative Western immunoblot procedure [14]. Briefly, clarified samples (10 µl) were added to 10 µl of sodium dodecyl sulfate polyacrylamide gel electrophoresis (SDS-PAGE) sample buffer, bringing the final volume to 1/10^th^ original culture volume. The total of 20 µl of each sample and buffer was electrophoresed on SDS-PAGE gels and transblotted onto 0.2 μm Polyvinylidene fluoride (PVDF) membranes (Billerica, MA). Control samples of purified TSST-1 were used as quantification standards, ranging from 10 µg/lane to 0.1 µg/lane. Subsequently, Western immunoblots were developed with hyperimmune antibodies against TSST-1 (Toxin Technologies, Sarasota, FL), then alkaline phosphatase-conjugated anti-rabbit IgG (Sigma-Aldrich), and finally substrate [14]. The color reactions were visualized by using NIH program ImageJ for quantitative comparison to purified TSST-1 samples [14]. The standard curve generated from purified TSST-1 typically gave R^2^ values of greater than 0.95, consistent with the reliability of this technique to be used quantitatively.

TSST-1 production by strain MNPE was quantified by a double immunodiffusion procedure [17]. Briefly, clarified samples as described above were serially diluted two-fold and reacted against hyperimmune rabbit serum against TSST-1 in double immunodiffusion slides. The last double immunodiffusion well to show visible precipitin arcs were compared to the lowest concentration of purified TSST-1 to react comparably (0.6 µg/ml original culture fluid) to determine amount of TSST-1 present in test samples.

Staphylococcal α-toxin concentrations were determined through lysis of rabbit erythrocytes in a slide assay [32]. Briefly, 30 µl of washed, packed rabbit erythrocytes were added to 0.85% agarose melted in phosphate-buffered saline (PBS, 0.005 M NaPO_4_, 0.15 M NaCl). This mixture was added to microscope slides (4 ml/slide). Upon solidifying, 20 µl of test samples were added to 4 mm wells punched into the agarose-erythrocyte slides. The slides were incubated at 37°C in the presence of 5% CO_2_ for 6 h. The area of each test sample was compared to the areas of known concentrations of purified α-toxin.

Cultures of a human normal vaginal isolate of *L. crispatus* were subjected to the same 9 hour assay as used to determine peptide antimicrobial effects on *S. aureus*. CFUs/ml were determined by dilution plate counts on Todd Hewitt agar (BD). The pHs of overnight broth cultures were also determined with use of a standard pH meter.

### Exotoxin-synthesis-inhibiting peptides

Peptides for use in all studies were either synthesized and purified to homogeneity by the University of Minnesota Microchemical Facility, or in the case of defensin peptides, were purchased from Sigma-Aldrich, St. Louis, Mo. All peptides were diluted into phosphate-buffered saline (PBS, 0.005 M NaPO_4_, 0.15 M NaCl) to concentrations ranging from 5.0 to 5×10^−6^ µg/ml. The composition of all peptides is shown in Table 1.

**Table 1:** Peptides tested for exotoxin synthesis inhibition activity.

Peptide Name	Amino Acid Sequence (% + charged amino acids)	Peptide Source
Hbg-1	SFPTTKTYFPHFDLSHGSAQVK (18%)	University of Minnesota
Hbg-2	STKPFFYFLHTQVKASPTSHDG (18%)	University of Minnesota
Hbg-1 attached to agarose bead	SFPTTKTYFPHFDLSHGSAQVK-agarose beads (18%)	University of Minnesota
SP-1	SFPTTATYFPAFDLSAGSAQVA (0%)	University of Minnesota
SP-2	SKPKKKTYKPHKDLSHGSAKKK (50%)	University of Minnesota
SP-3	SKPKKKKYKPHKKKSHKSAKKK (70%)	University of Minnesota
HNP-1	ACYCRIPACIAGERRYGTCIYQGRLWAFCC	Sigma-Aldrich
HNP-2	CYCRIPACIAGERRYGTCIYQGRLWAFCC	Sigma-Aldrich

### Human vaginal epithelial cell (HVEC) toxicity studies with exotoxin-synthesis-inhibiting peptides, and effect on interleukin 8 (IL-8) production by HVECs in response to *S. aureus* MN8

Overnight cultures of *S. aureus* MN8 were diluted to 10^9^ CFU in 1 ml, centrifuged 14,000×g for 5 min, and restored to up in 1 ml PBS. HVECs were propagated in Keratinocyte Serum-Free Medium (KSFM) (Gibco, Invitrogen, Carlsbad, CA) in the presence of 1% Fungizone (Thermo Scientific) and 1% penicillin-streptomycin (Gibco) in 96 well microtiter plates at 37°C and 7% CO_2_ until nearly confluent. Subsequently, the medium was changed to KSFM without antibiotics, and the cells were incubated for an additional 24 h. Then, the HVECs in triplicate were incubated for 6 h, at 37°C and 7% CO_2_, in the presence of anti-infective peptides (50% and 70%) alone (5 µg/ml or 50 µg/ml) for toxicity studies or with anti-infective peptides (5 µg/ml of 50% and 70%) and 1×10^4^ CFU/ml *S. aureus* MN8. After the incubation period, the supernates were removed and assayed for IL-8 production via Quantikine ELISA kit (R&D Systems) as a measure of inhibition of *S. aureus* production of pro-inflammatory chemokines. Previously, we have shown that both purified TSST-1 and *S. aureus* MN8 induce HVECs to up-regulate production of chemokines including IL-8 after 6 h incubation [33]. HVEC wells treated with peptides were also treated with 0.4% Trypan Blue (Sigma Aldrich) and viewed under an inverted microscope for determination of HVEC viability.

## Results

### Hemoglobin α-chain peptide (Hbg-1) inhibits TSST-1 production but not *S. aureus* MN8 growth

**Figure 1:**
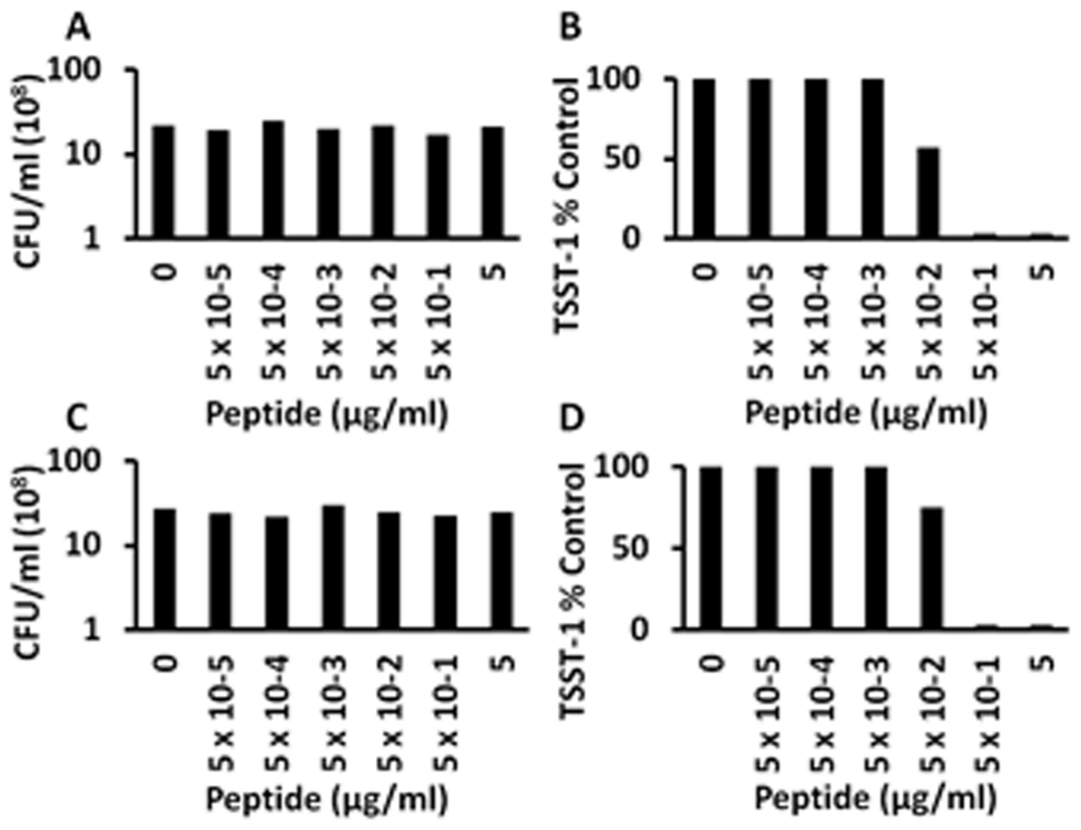
Exotoxin-synthesis-inhibition by hemoglobin (Hbg) peptide-1 (native peptide (A, B) and Hbg-2 (peptide with scrambled sequence; C,D) effects on *S. aureus* MN8 growth and TSST-1 production. *S. aureus* MN8 cultures were incubated at 37°C for 9 h with shaking 200 revolutions per minute in the presence of indicated amounts of anti-infective peptides. Subsequently, CFUs/ml were determined by plate counts, and TSST-1 amounts were quantified by Western immunoblotting.

We have previously shown that the α-globin chain of human hemoglobin inhibits TSST-1 production, without affecting *S. aureus* growth, through effects on SrrA/B and AgrA/C two-component systems [14]. Others have shown the hemoglobin peptides may inhibit bacterial growth, particularly gram-negative organisms, as hemacidins [34], [35]. With use of this information, we scanned the α-globin chain for a peptide that was previously considered a potent hemacidin for gram-negative bacteria and simultaneously cationic; we identified Hbg-1 (Table 1) as a hemacidin peptide containing 18% positively charged amino acids. This peptide did not inhibit the growth of *S. aureus* MN8 at any concentration tested (Fig. 1A). However, Hbg-1 inhibited TSST-1 production completely at 5.0 and 5×10^−1^ µg/ml (Fig. 1B) but lost activity at lower peptide concentrations. The activity to inhibit TSST-1 production was comparable on a molar basis to that observed previously for the activity of intact α-globin chains [14]. These experiments have been replicated on multiple days with similar results.

We were interested in whether or not the 3-dimensional shape of the peptide or its charge only was the important determinant of activity. We therefore scrambled the Hbg-1 peptide and assayed the new peptide (Hbg-2) for activity. Hbg-2 was not antimicrobial at any concentration tested (Fig. 1C), but the peptide was comparably active as Hbg-1 for inhibition of TSST-1 production (Fig. 1D). These data suggest that the exotoxin inhibition activity of the Hbg peptides was primarily related to charge properties. These experiments again have been replicated with separate experiments being performed on different days.

**Figure 2:**
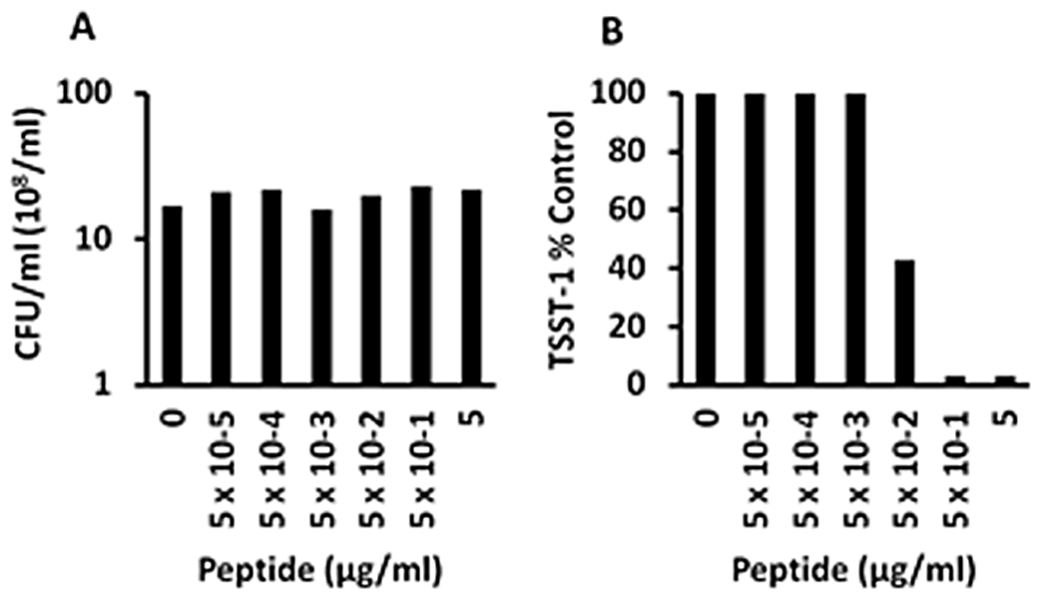
Exotoxin-synthesis-inhibition by hemoblobin (Hbg) peptide-1 effects on *S. aureus* MN8 growth and TSST-1 production when peptide was coupled to agarose beads. *S. aureus* MN8 cultures were incubated at 37°C for 9 h with shaking 200 revolutions per minute in the presence of indicated amounts of anti-infective peptides. Subsequently, CFUs/ml were determined by plate counts, and TSST-1 amounts were quantified by Western immunoblotting.

We performed one additional test of Hbg-1, evaluating whether the peptide would be active in inhibiting TSST-1 production when immobilized on agarose beads. If charge was the principal determinant of activity, then the immobilized peptide was hypothesized to retain activity. The Hbg-1 peptide, immobilized to agarose beads was not antimicrobial (Fig. 2A) but exhibited TSST-1 inhibition activity comparable to Hbg-1 not immobilized on beads (Fig. 2B). This experiment has been replicated multiple times on different days. These studies are important since they suggest that the Hbg peptides may be covalently attached to medical devices that can used in people without loss of exotoxin-synthesis-inhibition.

### Synthetic peptides (SP-1, SP-2, and SP-3) inhibit TSST-1 production dependent on percentage of positively-charged amino acids

**Figure 3:**
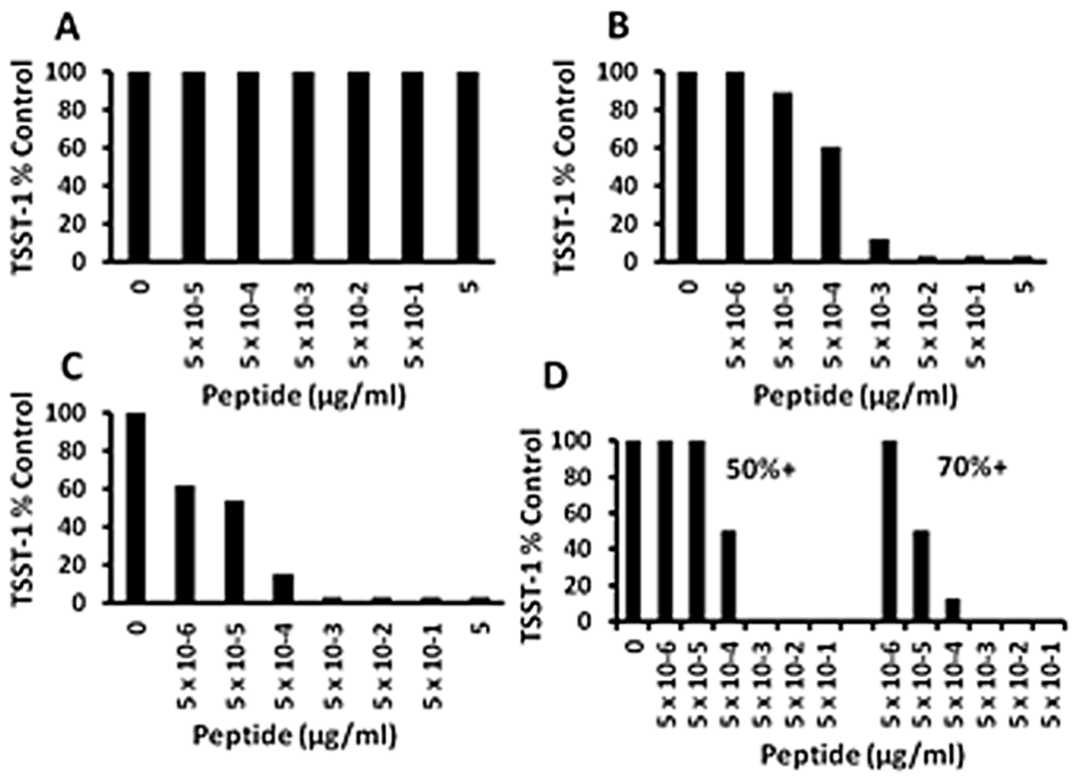
Exotoxin-synthesis-inhibition by hemoglobin-like synthetic peptide (A; SP-1; 0% positively-charged amino acids), SP-2 (B; SP-2; 50% positively-charged amino acids), and (C; SP-3; 70% positively-charged amino acids) effects on *S. aureus* MN8 TSST-1 production; SP-2 and SP-3 effects on *S. aureus* MNPE TSST-1 production (D). *S. aureus* MN8 and MNPE cultures were incubated at 37°C for 9 h with shaking 200 revolutions per minute in the presence of indicated amounts of anti-infective peptides. Subsequently, TSST-1 amounts were quantified by Western immunoblotting for MN8 and double immunodiffusion for MNPE.

Because of the TSST-1 inhibition activity of the above tested peptides, and data suggesting the activity resulted from the presence of positively charged amino acids, we tested Hbg-1 peptides that were modified to increase the number of positively-charged amino acids to include 0%, 50%, and 70% (Fig. 3 A,B,C). The peptide (SP-1), which lacked positive-charged amino acids, also lacked antimicrobial (data not shown) and TSST-1 inhibition activity when tested with either MN8 or MNPE (Fig. 3A shows data for MN8). In contrast, both highly positively-charged peptides (50% and 70%) showed the greatest TSST-1 inhibition activity when tested against either MN8 (Fig. 3B, C) or MNPE (Fig. 3D), compared to all other peptides tested, without affecting bacterial growth (Data not shown). SP-3 (70%) inhibited TSST-1 production by ≥50% at concentrations of 5×10^−5^ µg/ml or greater. This represents a 3-log improvement in TSST-1 inhibition compared to the original Hbg-1 peptide shown in Fig. 1B. These experiments have been replicated multiple times.

**Figure 4:**
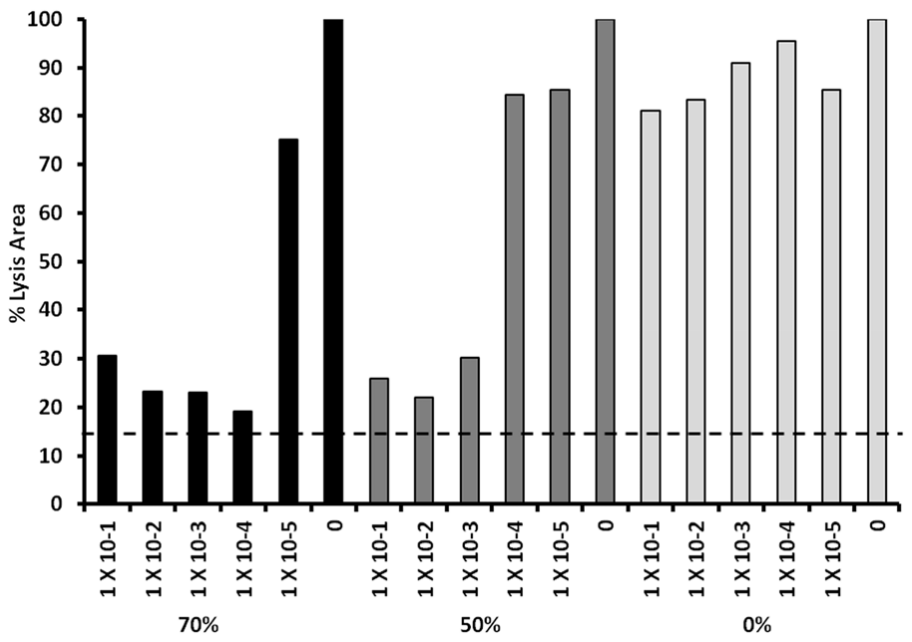
Exotoxin-synthesis-inhibition by hemoglobin-like synthetic peptides SP-1 (0% positively-charged amino acids), SP-2 (50% positively-charged amino acids), SP-3 (70% positively-charged amino acids) effects on *S. aureus* MNPE α-toxin production. *S. aureus* MNPE cultures were incubated at 37°C for 9 h with shaking 200 revolutions per minute in the presence of indicated amounts of anti-infective peptides. Subsequently, α-toxin amounts were quantified by rabbit erythrocyte lysis. The dashed line indicates measured area of wells without α-toxin lysis.

We also evaluated the effect of SP-1, SP-2, and SP-3 for effects on production of α-toxin by *S. aureus* MNPE (Fig. 4). SP-1 exhibited no inhibition of α-toxin production, whereas SP-2 showed inhibition at 5×10^−3^ µg/ml and SP-3 showed inhibition at 5×10^−4^ µg/ml, comparable to doses required for inhibition of TSST-1 production. These data show that the activity of the synthetic peptides is not limited to effects on TSST-1.

### HNP-1 and HNP-2 defensin peptides inhibit TSST-1 production by *S. aureus* MN8

**Figure 5:**
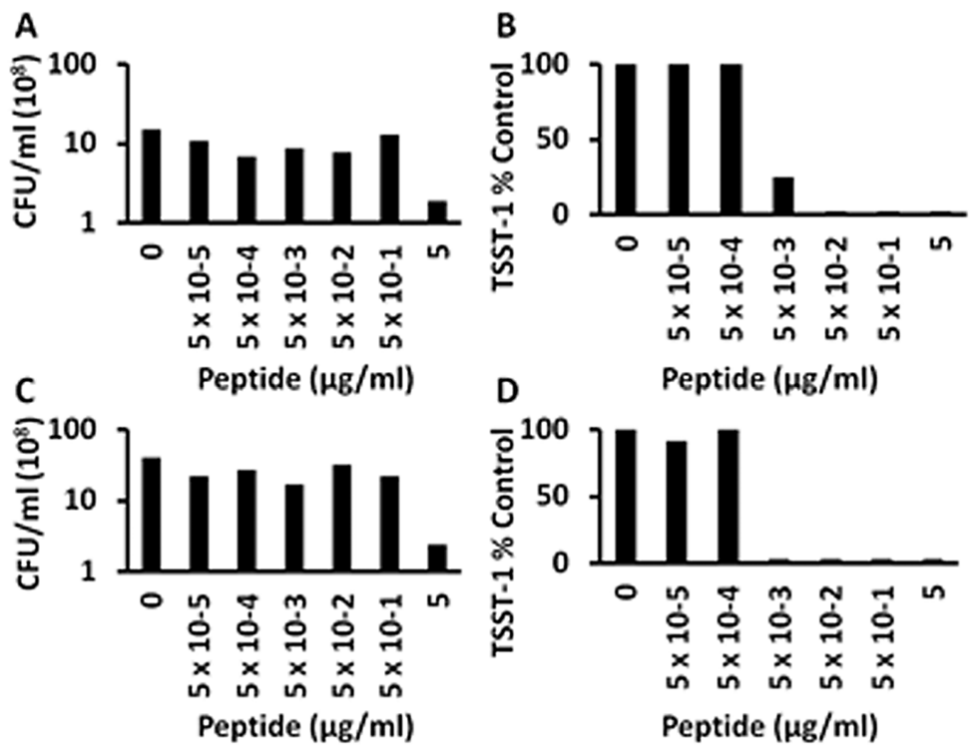
Antimicrobial activity and exotoxin synthesis inhibition activity, respectively, of (A+B) HNP-1 and (C+D) HNP-2 defensins. *S. aureus* MN8 cultures were incubated at 37°C for 9 h with shaking 200 revolutions per minute in the presence of indicated amounts of defensins. Subsequently, CFUs/ml were determined by plate counts, and TSST-1 amounts were quantified by Western immunoblotting.

Human defensin molecules are positively-charged peptides that have anti-staphylococcal activity [36]. We hypothesized that such peptides may have even greater ability to inhibit exotoxin production by *S. aureus* than antimicrobial activity. HNPs were weakly anti-staphylococcal with 5 µg/ml reducing *S. aureus* growth by 1 log (Fig. 5A,C). At all lower HNP concentrations, no antimicrobial activity was observed. In contrast, HNP-1 (Fig. 5B) and HNP-2 (Fig. 5D) were both highly inhibitory to production of TSST-1, with complete inhibition at doses as low as 5×10^−3^ µg/ml. These experiments have been replicated with similar results.

### Peptides do not inhibit *L. crispatus growth* and are not toxic to human vaginal epithelial cells (HVECs)

We evaluated the effect of Hbg-1, Hbg-2, SP-1, SP-2, and SP-3 on growth of a normal vaginal flora microbe, *L. crispatus*, in the 9 h assay. No inhibition of growth was observed, nor was alteration in media pH observed compared to untreated controls (data not shown).

The SP-2 (50%^+^) and SP-3 (70%^+^) peptides (5 µg/ml and 50 µg/ml) were not cytotoxic, by Trypan blue dye exclusion, to HVECs in 6 h assays (data not shown).

### IL-8 production by HVECs is reduced upon incubation with *S. aureus* MN8 and SP2 and SP-3

**Figure 6:**
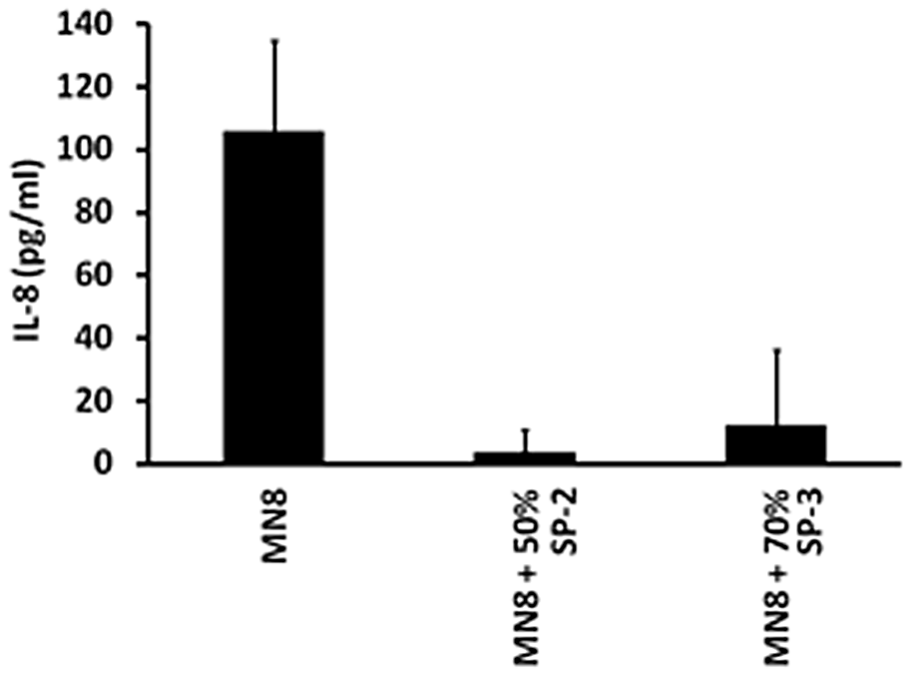
Prevention of IL-8 production by HVECs exposed to *S. aureus* MN8 and synthetic peptides. SP-2 (50% positively-charged amino acids) and SP-3 (70% positively-charged amino acids) at 5 µg/ml were incubated in quadruplicate in 96 well microtiter plates with human vaginal epithelial cells (HVECs) for 6 h. Subsequently, ELISA was used to measure IL-8 in supernates. Values represent means ± standard deviations.

We previously suggested that many microbes initiate disease on mucosal surfaces by stimulating epithelial cells to produce chemokines to attract immune cells into the tissue, with consequent disruption of barrier integrity. We tested the effect of SP-2 (50%^+^) and SP-3 (70%^+^) on the ability of *S. aureus* MN8 to stimulate chemokine production from HVECs. MN8 caused significant production of IL-8 (106±29 pg/ml) by HVECs after 6 h (Fig. 6). In contrast, incubation of HVECs with MN8 in the presence of 5 µg/ml of SP-2 or SP-3 synthetic peptides caused significant reductions in IL-8 production (3.6±7.2 pg/ml and 12.1±24.1, respectively) compared to treatment with MN8 alone (P = 0.001 and P = 0.002, respectively).

## Discussion

This manuscript addresses the ability of hemoglobin-derived peptides to inhibit the production of the superantigen TSST-1 and the cytolysin α-toxin without inhibiting *S. aureus* MN8 and MNPE growth. The basis for these studies is our prior observation that human hemoglobin α and β-globin chains inhibit exotoxin production without affecting microbial growth [14]. These hemoglobin chains inhibit exotoxin production through effects on two component systems on the *S. aureus* surface, including SrrA/B and possibly AgrA/C [14]. We have previously identified a series of molecules that comparably inhibit staphylococcal exotoxin production at sub-antimicrobial concentrations. These include the antibiotic clindamycin [17], GML [19], and the positively-charged polysaccharide chitosan [18].

When examined, there is no readily apparent shared property among the compounds, except all have significant effects on bacterial surfaces, either through their positive charges (hemoglobin chains and chitosan) or through their insertion into the plasma membranes (clindamycin and GML). Our study has identified a group of Hbg peptides, Hbg-based synthetic peptides, and HNPs that have TSST-1 and α-txoin inhibition activity, independent of antimicrobial activity. Except for HNPs at the highest concentration, none of the peptides studied in this manuscript exert antimicrobial activity at doses tested. All of the peptides with exotoxin inhibition activity are positively-charged, whether Hbg peptides, synthetic peptides, or HNPs. We hypothesize that all of these molecules, just as shown for the α-globin chain of hemoglobin, inhibit signal transduction through two component systems. These agents thus represent a novel class of molecules that can interfere with *S. aureus* function, despite lacking antimicrobial effects. It is potentially important that peptides studied in our research exert effects primarily dependent on charge, suggesting that the peptides can be immobilized on medical devices and still retain activity. This was observed in our study when Hbg-1 was immobilized on agarose beads.

Previous studies in rabbit models have demonstrated that pre-existing immunity to superantigens prevents not only serious illnesses but also colonization [27],[28], suggesting that even the ability of *S. aureus* to colonize depends on superantigen production. Cytolysins are also important in causation of human illnesses [37]. The major cytolysin, α-toxin, is also inhibited by α- and β-globin chains of hemoglobin [14]. These exotoxins have been suggested to lead to *S. aureus* colonization through an outside-in signaling mechanism whereby the exotoxins on mucosal surfaces trigger low-level inflammation, beginning with chemokine production from epithelial cells, which disrupts the protective mucosal barrier to infection [26], [38], [39].

Recently, multiple other small molecules have been identified by others that inhibit the production of exotoxins, including α-toxin, at doses that do inhibit the growth of *S. aureus* [40]–[42]. Interestingly, many of these small molecules, including solonamides and biaryl compounds exert their effects at least in part by interfering with the function of the Agr two-component system. The compounds interfere with α-toxin production while at the same time increasing production of protein A, as would be expected with effects on the Agr system. We previously showed that the α- and β-globin chains of hemoglobin inhibit exotoxin production while also increasing production of protein A. In the case of the hemoglobin peptides, at least two of the staphylococcal two component systems, SrrA/B and AgrA/C, were altered by the hemoglobin chains. The Hbg-1 and synthetic peptides studied in this manuscript also exhibit these same properties of inhibiting exotoxin production while at the same time increasing protein A production. Finally, we have recently identified coenzyme Q1 as an antistaphylococcal agent that inhibits exotoxin production at doses that do not inhibit bacterial growth [43]. Those studies suggest that two component systems are at least in part responsible for the observed exotoxin-synthesis-inhibition. Collectively, these data are important because they indicate there are likely to be families of important small molecules that inhibit exotoxin production while not inhibiting *S. aureus* growth. This is a desirable property for potential use of the molecules on human skin and mucosa, when applied to medical devices, cosmetics, or in foods, wherein it may be harmful to alter normal microbial flora, yet desirable to interfere with exotoxin production and associated diseases such as menstrual TSS.

Our unpublished studies indicate that *Lactobacillus species*, including *L. crispatus*, that are normal vaginal flora, do not depend on inflammation for colonization. Instead, these organisms secrete anti-inflammatory factors that appear necessary to prevent colonization by potential pathogens [44]–[46]. Thus, it is important that the present studies have shown that inhibition of *S. aureus* exotoxin production by cationic peptides occurs, and in absence of inhibition of *S. aureus* growth, likely will prevent *S. aureus* colonization. In contrast, lactobacilli, which do not depend on exotoxin production for colonization, are likely to be unaffected by the cationic peptides. Consistent with this hypothesis is the finding in this study that *L. crispatus* growth and production of lactic acid was unaffected by the cationic peptides. Finally, it is also significant that the peptides used in our studies were not cytotoxic for HVECs.
